# Comprehensive Molecular Analyses of Notch Pathway-Related Genes to Predict Prognosis and Immunotherapy Response in Patients with Gastric Cancer

**DOI:** 10.1155/2023/2205083

**Published:** 2023-01-24

**Authors:** Yinsen Song, Na Gao, Zhenzhen Yang, Sisen Zhang, Tianli Fan, Baojun Zhang

**Affiliations:** ^1^School of Basic Medical Sciences, Xi'an Jiaotong University, Translational Medicine Research Center, Zhengzhou People's Hospital, Zhengzhou, China; ^2^Translational Medicine Research Center, Zhengzhou People's Hospital, Zhengzhou, China; ^3^School of Basic Medical Sciences, Xi'an Jiaotong University, Zhengzhou People's Hospital, Zhengzhou University, Zhengzhou, China; ^4^Department of Pathogenic Microbiology and Immunology, School of Basic Medical Sciences, Xi'an Jiaotong University, Xi'an, China

## Abstract

Gastric cancer (GC) is a highly molecular heterogeneous tumor with unfavorable outcomes. The Notch signaling pathway is an important regulator of immune cell differentiation and has been associated with autoimmune disorders, the development of tumors, and immunomodulation caused by tumors. In this study, by developing a gene signature based on genes relevant to the Notch pathway, we could improve our ability to predict the outcome of patients with GC. From the TCGA database, RNA sequencing data of GC tumors and associated normal tissues were obtained. Microarray data were collected from GEO datasets. The Molecular Signature Database (MSigDB) was accessed in order to retrieve sets of human Notch pathway-related genes (NPRGs). The LASSO analysis performed on the TCGA cohort was used to generate a multigene signature based on prognostic NPRGs. In order to validate the gene signature, the GEO cohort was utilized. Using the CIBERSORT method, we were able to determine the amounts of immune cell infiltration in the GC. In this study, a total of 21 differentially expressed NPRGs were obtained between GC specimens and nontumor specimens. The construction of a prognostic prediction model for patients with GC involved the identification and selection of three different NPRGs. According to the appropriate cutoff value, the patients with GC were divided into two groups: those with a low risk and those with a high risk. The time-dependent ROC curves demonstrated that the new model had satisfactory performance when it came to prognostic prediction. Multivariate assays confirmed that the risk score was an independent marker that may be used to predict the outcome of GC. In addition, the generated nomogram demonstrated a high level of predictive usefulness. Moreover, the scores of immunological infiltration of the majority of immune cells were distinctly different between the two groups, and the low-risk group responded to immunotherapy in a significantly greater degree. According to the results of a functional enrichment study of candidate genes, there are multiple pathways and processes associated with cancer. Taken together, a new gene model associated with the Notch pathway may be utilized for the purpose of predicting the prognosis of GC. One potential method of treatment for GC is to focus on NPRGs.

## 1. Introduction

Gastric cancer (GC) is one of the most prevalent malignant tumors in the world [1]. GC was ranked as the fifth biggest cancer burden in the world, according to data from the World Health Organization (WHO) [2]. This was based on the estimated occurrence of 1 million cases worldwide [3]. As a result of the late detection of the disease at a more advanced stage, the mortality rate of gastric cancer is significant; for example, in 2020, it was 768,793, which places it as the fifth most prevalent cause of death due to cancer [4, 5]. Despite the fact that surgery is the primary treatment with the intention of curing the disease, 40%–60% of those patients who undergo resection surgery show disease relapse [6]. The prognosis is not good for these individuals, who have a survival rate of fewer than 10% over a period of 5 years [7, 8]. Patients with GC face an uphill battle when it comes to their prognosis because of intratumoral, interpatient, and intrapatient heterogeneity of the disease [9]. In addition, GC has a predisposition toward early metastasis, and the majority of these metastases are discovered in severe stages. This may be the single most important factor contributing to the high fatality rate of GC patients [10, 11]. As a result, it is essential to locate dependable predictors for the purpose of prognostic estimation, which might provide an incredible amount of guiding value to the administration of the GC. Patients suffering from GC would especially benefit from an improved prognostic prediction if a multiple-gene signature could be constructed.

The Notch signaling system is an intercellular signaling pathway that has been largely conserved throughout evolution. It is responsible for regulating cell proliferation and differentiation, determining cell fate decision, and participating in cellular activity in both embryonic and adult tissues [12, 13]. It is essential to have a good understanding of the structure of Notch proteins and the signaling pathways that are associated with them, since they are involved in the control of the promotion, proliferation, and development of cancer [14, 15]. Transmembrane glycoproteins make up Notch receptors, which range in the number from 1 to 4. Notch receptors have three distinct regions: an extracellular domain, a transmembrane domain, and an intramembrane or cytoplasmic region [16]. The extracellular domain is located outside of the cell. The activation of oncogenic signaling pathways makes aggressive GC harder to treat. One such pathway is the Notch signaling pathway. Notch signaling is an important mechanism in the process of the self-renewal of stem cells, the determination of cell fate and differentiation during embryonic and postnatal development, and the maintenance of adult cell homeostasis [17, 18]. Until now, it is not clear whether each Notch component acts as an oncogene or a tumor suppressor. This is a contentious issue. Researchers also focused on the connection between the Notch signaling pathway and stomach cancer [19, 20]. Although a rapidly expanding number of linked outcomes have been developed, conclusions are still debatable. For example, in contrast to conventional wisdom, researchers found that the expression of Notch 1 was lower in stomach cancers than that in normal tissue.

There is an increasing body of research studies suggesting that the tumor microenvironment (TME) plays an important part in the progression of tumors [21]. It is possible to divide solid tumors into two categories: immunologically hot tumors and cold tumors [22]. Cancer immunotherapy is successful in treating hot tumors, whereas the treatment is ineffective against cold tumors [23]. Cancers that are immunologically inert have a few mutations, a limited invasion of cytotoxic immune cells, and a substantial population of myeloid-derived suppressor cells [24, 25]. As a consequence, reactions seen in clinical trials involving immune checkpoint blockade (ICB) are poorer in immunologically cold tumors [26]. However, preliminary research has demonstrated that it is possible for cool tumors to become heated ones. Therefore, it is of the utmost importance to discover the comprehensive mechanism that lies at the root of immunologically cold tumors, as this would aid in the development of a method for bringing cold tumors up to temperature and turning them into hot tumors.

In this study, we built a predictive signature using genes related to Notch pathway-related genes (NPRGs), evaluated its utility for determining outcomes, diagnosis, treatment responses, and tumor immune infiltration of GC patients, and carried out internal verification. In addition, we accomplished functional enrichment analysis (GSEA) in order to investigate possible mechanisms.

## 2. Materials and Methods

### 2.1. Raw Data

The data on RNA sequencing and clinical information related to STAD patients were received from the TCGA data portal. For the purpose of developing the risk model, the TCGA-STAD cohort served as the “training cohort,” while the microarray data obtained from the GEO database was applied to the “validation cohort.” We collected RNA-sequencing data on the TCGA-STAD cohort using the UCSC Xena browser (https://xenabrowser.net/datapages/) in raw count format. We then normalized the data for subsequent analysis by using Deseq2 software. GSE84437 included 433 GC samples with available clinical information. Downloads of gene sets relevant to the human Notch pathway were received from the Molecular Signature Database (MSigDB), and a total of 428 genes were retrieved from seven different Notch-related pathways (Table S1).

### 2.2. Identification of Differentially Expressed genes (DEGs) in GC

Raw count data were first transformed into log2 form after being standardized with the transcript per million (TPM) method. The next step was the annotation of 19654 protein-coding genes. Limma, version 3.36.2 of the R package, was utilized in the determination of DEGs [27]. The detection of differentially expressed genes (DEGs) worthy of further investigation required both a log2 fold change (FC) of larger than one and an adjusted *P* value of less than 0.05.

### 2.3. Construction of the Prognostic Model by LASSO Cox Regression

To determine which candidate DEGs in the two discovery sets were most strongly related to patients' overall survival times, we used univariate analysis with a significance level of *P* < 0.05. In order to perform univariate Cox regression analysis, the “survival” R program was utilized [28]. Least absolute shrinkage and selection operator (LASSO) Cox regression, together with ten times of cross-validation, was utilized in order to arrive at the value for the penalty regularization parameter. The coefficient of each gene was decreased to zero by artificial means, which got rid of the connection that existed between the genes that were chosen and stopped the model from being overfit. Genes were chosen using a method called lamda.min, which stands for minimum deviance. In order to perform LASSO Cox regression analysis, the “glmnet” R program was utilized. Multivariate Cox regression analysis was used to create the coefficients for each gene, and then, the prognostic risk-score model was constructed using those coefficients. In order to carry out the multivariate Cox regression analysis, the “survminer” R program was utilized [29]. The risk score for each patient was determined using the risk-score model based on the expression of each gene that was found. After that, the model of risk scores was utilized to determine the prognosis of GC patients. The TCGA cohort served as the training set, whereas the samples from the GEO: GSE84437 project served as the test set.

### 2.4. Building Predictive Nomograms and Analyzing Gene Set Enrichment for Functional Relevance

The “rms R package” was used in order to construct the nomogram and the calibration plot [30]. Gene set enrichment analysis (GSEA) was applied to GC patients in order to uncover associated pathways. It was determined that an enriched gene set was statistically significant if it had a false discovery rate of less than 0.25 percent and a nominal *P* value that was lower than 0.05 percent.

### 2.5. Principal Component Analysis (PCA)

In the field of computer vision, PCA is a method that is frequently used for dimensionality reduction and feature extraction [31]. In order to study the potential differences that may exist between high-risk and low-risk groups, the “scatterplot3d” R tool was used [32].

### 2.6. Immune Feature Analysis

A brand new deconvolution algorithm known as CIBERSORT was used, which is based on linear support vector regression [33]. Taking into consideration the important roles that immune cells play in TME, the CIBERSORT program was applied to determine the scores of 22 immune cells in each tumor sample. Using the ggplot2 R tool, all of the results were displayed on stacked graphs, heatmaps, and box plots, respectively [34]. In addition, the Wilcoxon rank-sum test was utilized in order to conduct an analysis of disparate scores exhibited by these immune cells that were obtained from TCGA datasets.

### 2.7. Pathway Analysis

The DEGs of low-risk and high-risk groups were compared using the “edgeR” tool of the R computer language. In this investigation, we compared the DEGs of the two groups. This was performed in order to perform functional annotation from the GO for DEGs. The KEGG database performs an analysis of metabolic pathways. After that, GSEA was then performed to reveal signaling pathways and BPs in which differentially expressed genes were enriched between high-risk and low-risk subgroups.

### 2.8. Statistical Analysis

All analyses used in this study were performed by using R software (version 3.5.1, Boston, Massachusetts, USA). The Kaplan–Meier curve, which was examined by the log-rank test, was utilized in the investigation of the connection between genes associated with the Notch pathway and overall survival. For the purpose of determining the sensitivity and specificity of the prognostic prediction model, time-dependent ROC curves were utilized. The performance of the nomogram was evaluated using the c-index and the calibration curve. The nomogram was produced using the regression coefficients that were derived from the Cox analysis. Statistical differences between the two groups were examined using the Wilcoxon test. When the *P* value was less than 0.05, statistical significance was considered.

## 3. Results

### 3.1. Identification of DEGs between Normal Specimens and GC Tissues

**Figure 1:**
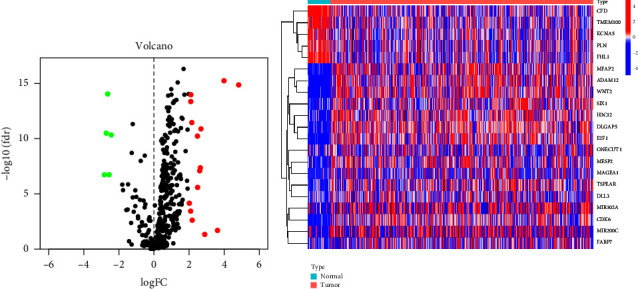
Differentially expressed NPRGs between GC specimens and nontumor specimens shown in the (a) volcano map and (b) heatmap.

Seven gene sets associated with the Notch pathway were obtained from the MSigDB database. Using data received from TCGA-STAD, we were able to obtain information on the linked gene expression of GC. The “limma” R program was applied in order to locate genes that displayed differential expression levels. TCGA-STAD was used to analyze the differential expression of 95 distinct NPRGs. As shown in Figures 1(a) and 1(b), a total of 21 DEGs were obtained: 16 genes (MIR302A, MIR200C, DLGAP5, E2F1, CDK6, FABP7, MFAP2, TSPEAR, MESP2, SIX1, H3C12, ONECUT1, DLL3, ADAM12, WNT2, and MAGEA1) were significantly upregulated and 5 genes (KCNA5, TMEM100, CFD, PLN, and FHL1) were significantly downregulated.

### 3.2. Establishment of the Prognostic Notch Pathway-Related Gene Signature

**Figure 2:**
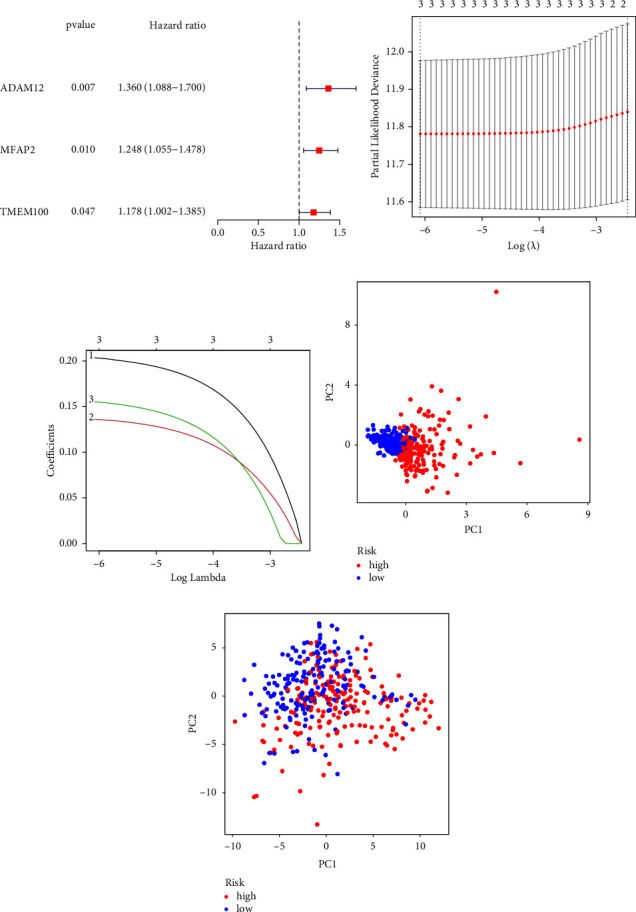
Construction of a prognostic signature in GC from TCGA. (a) Hazard ratios for three differentially expressed NPRGs that were implicated in overall survival plotted on a forest plot. (b) Three-fold cross-validation for tuning parameter selection in the LASSO model. (c) Profiles of differentially expressed NPRGs using the LASSO coefficient. The value determined using a three-fold cross-validation is indicated by the dashed line. (d) Principal component analysis based on NPRGs in GC. (e) In order to differentiate tumor samples from normal ones in the TCGA cohort, principal component analysis was performed based on a risk score. Patients who were considered to have a high risk were represented by the group that was colored green, while patients who were considered to have a low risk were represented by the group that was colored red.

**Figure 3:**
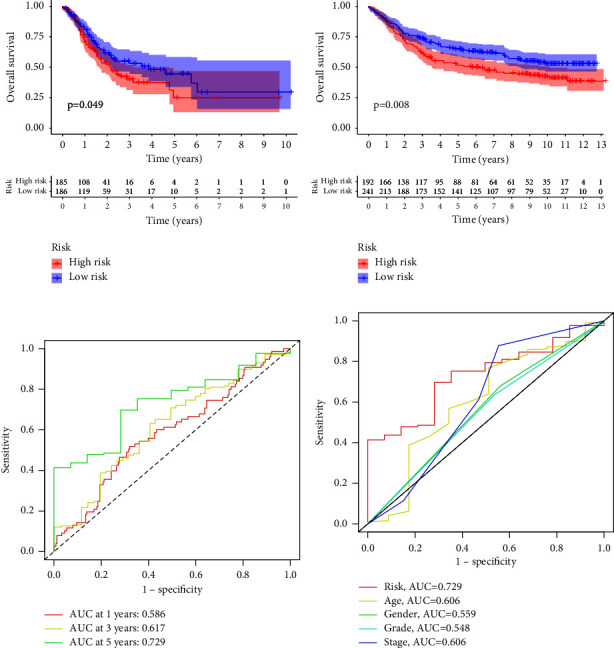
Validation of the risk-score model. (a, b) The survival value of the risk score was demonstrated in TCGA datasets and GSE84437. (c) Validation of the prognostic values of the index in TCGA datasets using a ROC curve. (d) ROC assays for both clinical factors and risk scores.

**Figure 4:**
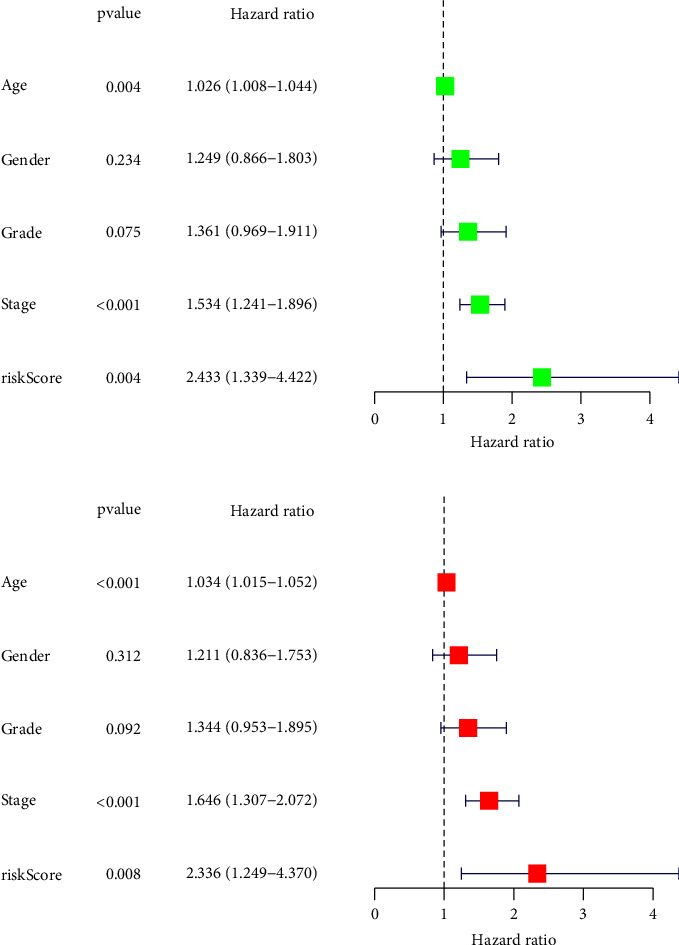
(a) Univariate and (b) multivariate analysis of overall survival in GC patients from TCGA datasets.

For the purpose of predicting overall survival in patients from TCGA datasets, LASSO and Cox assays were employed to evaluate a gene signature connected with 3 Notch pathways, and the formula calculating the risk score was as follows: ADAM12 expression ^*∗*^0.2035 + MFAP2 expression ^*∗*^0.1361 + TMEM100 expression ^*∗*^0.1554 (Figures 2(a)–2(c)). To clearly differentiate GC samples, the risk-score model was applied (low or high-risk) (Figures 2(d) and 2(e)). According to the findings of patient survival, those patients who had a low-risk score had a greater survival rate than those patients who had a high-risk score (Figure 3(a)). In addition, these findings were reexamined and shown to be consistent for GSE84437 datasets (Figure 3(b)). According to the results of a time-dependent ROC analysis, the Notch pathway-related gene signature had a diagnostic accuracy of 0.586 after one year, 0.617 after three years, and 0.729 after five years (Figure 3(c)). The area under the ROC curves (AUC) demonstrated that the risk score (AUC = 0.729) had a better prognostic value than a single indicator, such as age (AUC = 0.606), gender (AUC = 0.559), grade (AUC = 0.548), and stage (AUC = 0.606) (Figure 3(d)). Cox survival studies were carried out so that we could find out whether or not the risk score was an independent factor for determining the outcome of GC. According to the results of a univariate study, the clinical stage and the risk score were associated with the patients' likelihood of survival with GC (Figure 4(a)). Furthermore, multivariate analysis revealed that a patient's risk score was an independent predictor of a poor prognosis for GC (HR = 2.336, 95% CI: 1.249–4.370). (Figure 4(b)).

### 3.3. Association between Clinicopathological Characteristics and Risk Scores

**Figure 5:**
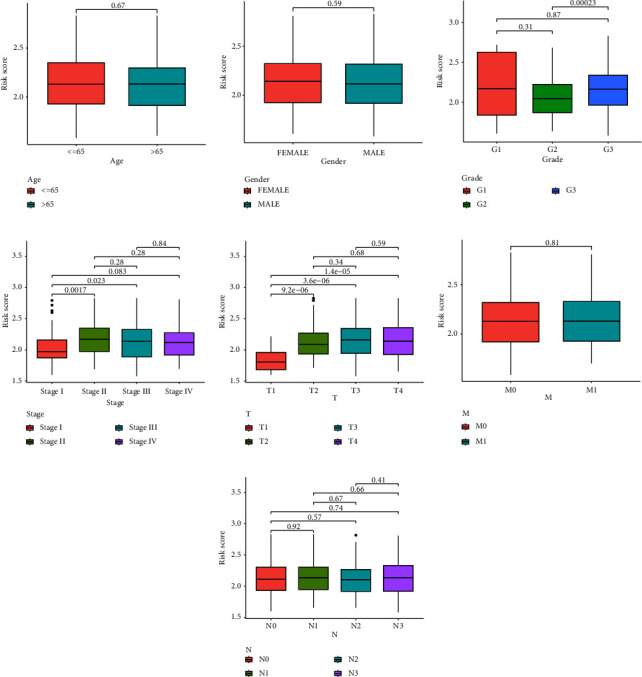
Indications of a link between the risk model and patient features. Correlation assays of the risk score with (a) age, (b) gender, (c) grade, (d) stage, (e) *T* stage, (f) *M* stage, and (g) *N* stage.

The potential link between the risk score and clinicopathological features was then investigated. We discovered no correlation between the risk score and either age or gender (Figures 5(a) and 5(b)). In addition, we observed that a higher risk score was associated with the advanced grade (Figure 5(c)), clinical stage (Figure 5(d)), and *T* stage (Figure 5(e)). However, there was not a distinct difference in the risk score between the *M* and *N* stage (Figures 5(f) and 5(g)).

### 3.4. Construction of a Nomogram for Predicting Survival

**Figure 6:**
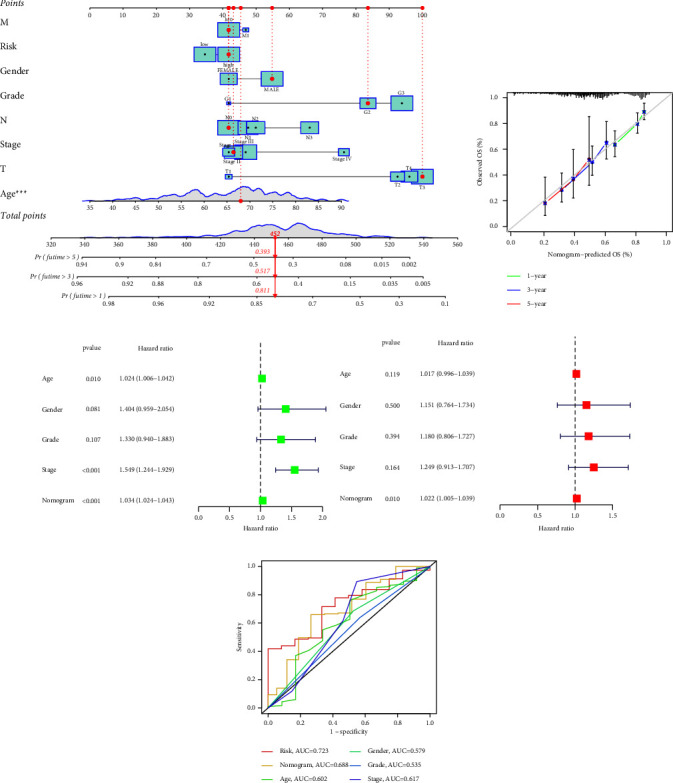
The development of the nomogram with the risk score. (a) A nomogram predicting survival. (b) A calibration plot for prediction. (c and d) Univariate and multivariate analyses of the nomogram with the risk score. (e) The nomogram with the risk score.

To better predict OS for GC data, a nomogram was constructed using age, gender, grade, pathological stage, *T* stage, *M* stage, and *N* stage information, in addition to a predictive risk-score model (Figure 6(a)). The nomogram's ability for the prediction of the overall survival of GC patients was demonstrated by calibration curves drawn at 1, 3, and 5 years (Figure 6(b)). Cox assays illustrated that the nomogram is an independent prognostic indicator for GC patients (Figures 6(c) and 6(d)). The nomogram was more predictive than a single indicator, as shown by AUC (Figure 6(e)).

### 3.5. Gene Set Variation Analysis (GSVA)

**Figure 7:**
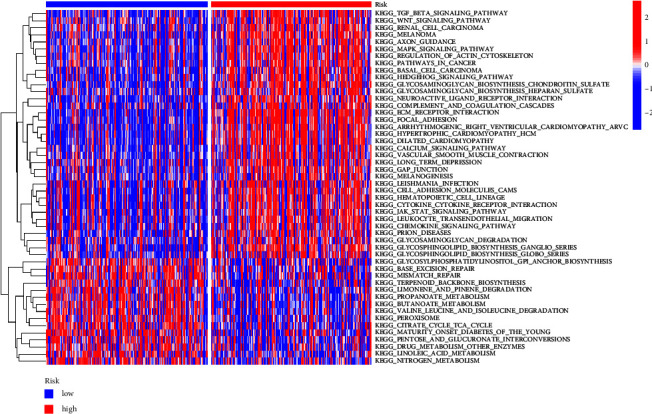
Comparison of GSVA enrichment for groups with low and high-risk scores as shown in a heatmap.

To investigate biological activities exhibited by the two groups, GSVA enrichment was carried out with the gene sets of “c2.cp.kegg.v7.2,” which were obtained from the Molecular Signature Database (MSigDB). Interestingly, we found that many tumor-related were enriched in the high-risk score, such as TGF_BETA_SIGNALING_PATHWAY, WNT_SIGNALING_PATHWAY, and KEGG_MAPK_SIGNALING_PATHWAY (Figure 7). Our findings suggested that the above genes may be involved in tumor progression via regulating several different tumor-related pathways.

### 3.6. Relationships between the Gene Signature and Immune Cells

**Figure 8:**
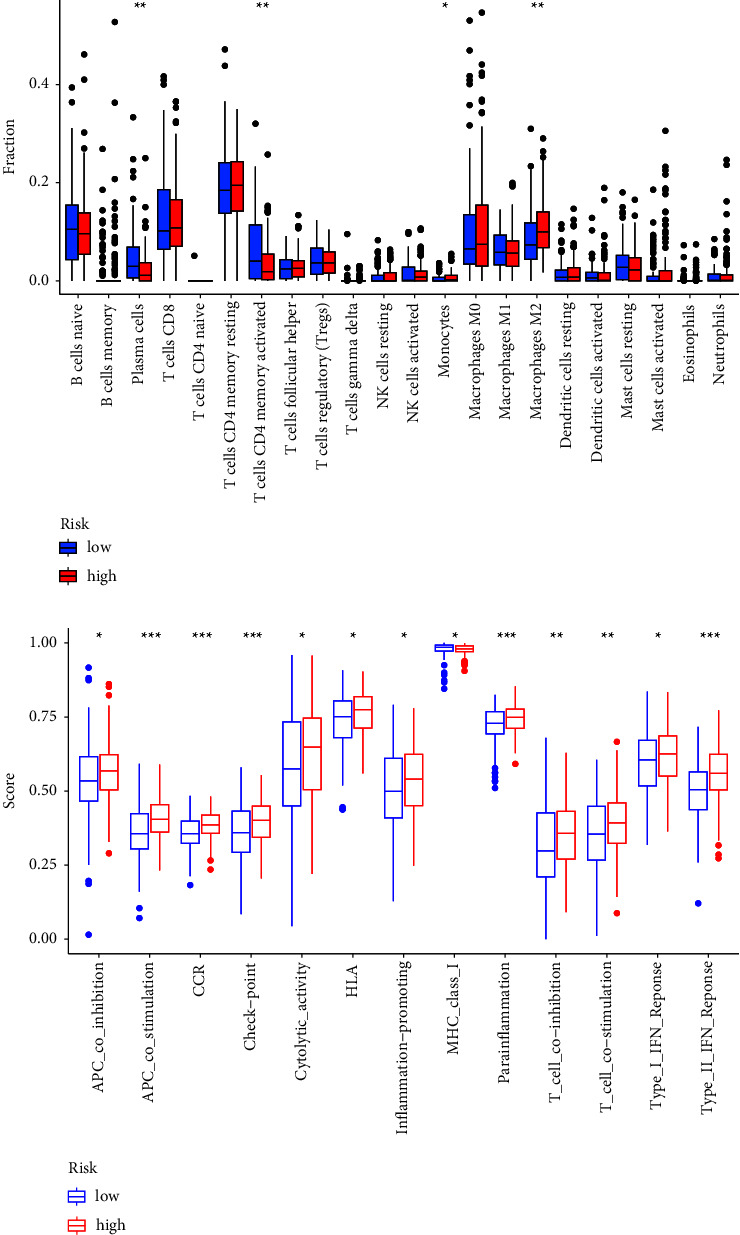
Modeling immunotherapy risk. (a) Changes in immune cell infiltration between people with low and high-risk scores. (b) Cases with a high-risk score and those with a low-risk score have different observable functions associated with immune system control.

We estimated the presence of 22 immune cell types in the TCGA cohort.  Figure 8(a) displays the substantial difference in the presence of four types of immune cells between cases in the low-risk group and cases in the high-risk group (plasma cells, T cells CD4 memory activated, monocytes, and macrophages M2). Moreover, APC_co_inhibition, APC_co_stimulation, CCR, Check-point, Cytolytic_activity, HLA, Parainflammation, T_cell_co-inhibition, T_cell_co-stimulation, Type_I_IFN_Response, and Type_II_IFN_Response were also activated in the high-risk group, indicating that it is possible that immunotherapy will be effective for people in the high-risk group who have immune suppression (Figure 8(b)).

### 3.7. Enrichment Analyses

**Figure 9:**
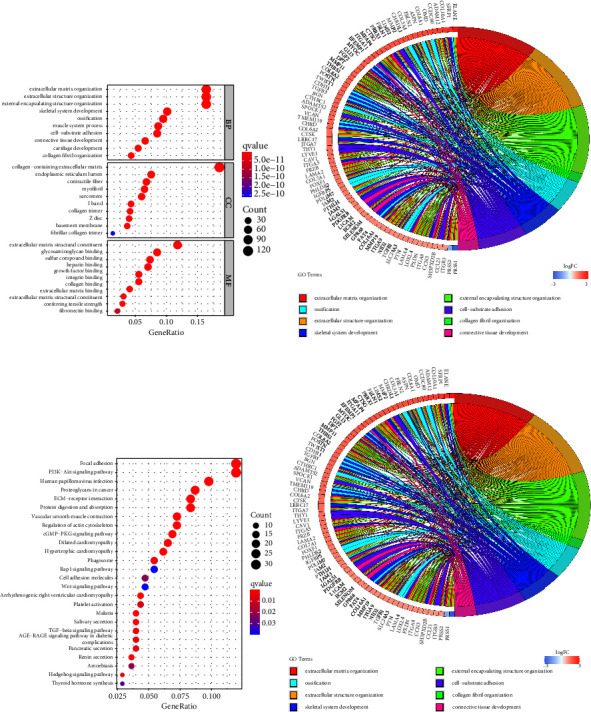
Comparing the TCGA cohort's two risk categories from the lens of functional analysis based on DEGs. (a, b) GO enrichment. (c, d) KEGG pathways.

To isolate DEGs, we used the “limma” R package and filtered for FDR 0.05 and |log2FC| > 1. These steps were taken to delve deeper into how the risk model's categorization of individuals into subgroups affects gene function and pathway analysis. We found that there were a total of 686 DEGs that existed between the low-risk and high-risk groups in TCGA datasets. There were 627 upregulated genes and 58 downregulated genes in the high-risk group. As shown in Figures 9(a) and 9(b), we found that 627 genes were mainly associated with extracellular matrix organization, extracellular structure organization, skeletal system development, endoplasmic reticulum lumen, contractile fiber, myofibril, extracellular matrix structural constituent, glycosaminoglycan binding, and sulfur compound binding. In addition, the results of KEGG assays revealed that 627 genes were mainly associated with focal adhesion, PI3K-Akt signaling pathway, human papillomavirus infection, proteoglycans in cancer, and ECM-receptor interaction (Figures 9(c) and 9(d)).

## 4. Discussion

GC is one of the most prevalent malignancies worldwide [35]. The most recent statistics available on the disease indicated that GC is currently ranked as the world's second most prevalent cause of death from cancer-related causes [36, 37]. The majority of GC is caused by *Helicobacter pylori'*s complicated interplay with the host's components. According to the findings of a number of studies, a number of environmental factors, including trace elements, are thought to be contributors to the development of stomach cancer [38, 39]. Even with the breakthroughs that have been made in diagnosis and therapy over the course of the last few years, the primary therapeutic option for GC patients remains surgery. The prognosis for individuals with GC is still not favorable due to the fact that a significant number of patients are still initially diagnosed at an advanced stage. As a result, it is of the utmost significance to look for promising prognostic indicators for early diagnosis and innovative therapy targets.

Notch was identified for the first time in 1917 and was given its name after the mutation that was found to cause malformations in the wings of flies [40]. The Notch signaling pathway is an example of a chained signaling pathway. It is made up of ligands, receptors, and DNA binding proteins farther down the chain [41]. Notch1 and Notch2 are two different types of receptors, and jagged1 is considered to be a ligand [42]. In addition, the roles of the Notch signaling pathway in GC have been verified and proved. It has been observed that the Notch and mTOR signaling pathways are frequently activated in human stomach cancer, which contributes to the proliferation of cells [43, 44]. It is possible that a viable therapeutic strategy for treating GC would involve targeting these pathways in combination with one another. According to the findings of Yang et al., the Notch signaling pathway may play a key role in the course of GC as well as the prognosis of the disease by regulating the function of CD4+CD25+CD127-dim/- regulatory T cells and *T* helper 17 cells [45]. The findings brought attention to the significant functions that the Notch pathway plays in the evolution of GC. As a result, we were curious as to whether or not a unique prognostic model that was based on NPRGs could be utilized in the process of forecasting the prognosis of patients who had GC. In this study, using TGCA datasets, we were able to obtain a total of 21 NPRGs with differential expression in GC. The expression of ADAM12, MFAP2, and TMEM100 was then used to establish a diagnostic signature for the disease. Based on the TCGA database, this prognostic model displayed an outstanding performance for operating system prediction. According to comprehensive research, the Notch pathway-related prognostic model was shown to be an independent prognostic indicator when other clinical parameters were taken into account. Subsequently, a model comprising nine NPRGs was effectively verified as a predictive factor for an independent GEO dataset. This was accomplished after the model was initially developed. Integration with a subset of clinicopathological characteristics in a risk-assessment nomogram further enhanced the predictive value of this prognostic risk-score model. This resulted in the nomogram having a higher predictive capacity. All of these data pointed to the fact that the Notch pathway-related prognostic model has the potential to serve as an efficient marker for GC prognostic prediction.

The landscape of cancer treatment is now being altered as a result of the application of immunotherapy to the treatment of a variety of malignancies [46, 47]. For instance, inhibiting the interaction between PD-1 and PD-L1 can restore the function of effector T cells, allowing them to perform their intended role of eliminating tumor cells more effectively. The level of PD-L1 that was expressed in a patient's tumor is the most important element in identifying whether or not they are a candidate for PD-1/PD-L1 axis immunotherapy. However, in practice, many PD-L1-positive patients have a poor response to PD-1/PD-L1 axis treatment, whereas some PD-L1-negative patients have an unexpectedly excellent response. Our study showed that high-risk patients with up-regulated immunological checkpoints had a worse response to immunotherapy, which was the result that kept popping up. However, the existence of immune cell infiltration may be a predictor of how effectively immunotherapy works, as individuals in the low-risk subgroup who had higher levels of immunological/inflammatory activity were more likely to benefit from the treatment.

However, this study also had certain limitations. First, the bioinformatic research for this work was only performed on publicly available datasets. Next, we need to make sure that the findings of this investigation are accurate by using clinical participants in a prospective study design. Second, the three genes that make up the prognostic signature are all known to be risk factors in patients diagnosed with GC. Their downstream molecular pathways require additional investigation through functional tests in order to discover potential novel treatment targets. Overall, our gene profile that is associated with the Notch pathway has a fair chance of accurately predicting the immunotherapy response; however, this hypothesis will need to be verified in the future using clinical studies that are carefully planned.

## 5. Conclusion

Overall, a strong Notch pathway-related prognostic model was created, and the characteristics of the tumor immune milieu were investigated; our findings could be beneficial to the diagnosis and treatment of patients with GC.

## Data Availability

The data used to support the findings of this study are included in the article.

## Supplementary Materials

Supplementary Materials Table S1: all names of Notch-related genes.
