# Supplementary material for: Comprehensive Molecular Analyses of Notch Pathway-Related Genes to Predict Prognosis and Immunotherapy Response in Patients with Gastric Cancer
Source: J Oncol. 2023 Jan 24;2023:2205083. doi: 10.1155/2023/2205083 (PMC9889149; doi:10.1155/2023/2205083)
Supplement: Supplementary Materials — Table S1: all names of Notch-related genes. [file 2205083.f1.docx]

Table S1 All names of Notch-related genes.

Gene names

ADAM17

APH1A

CIR1

CREBBP

CTBP1

CTBP2

DLL1

DLL3

DLL4

DTX1

DTX2

DTX3

DTX3L

DTX4

DVL1

DVL2

DVL3

EP300

HDAC1

HDAC2

HES1

HES5

JAG1

JAG2

KAT2A

KAT2B

LFNG

MAML1

MAML2

MAML3

MFNG

NCOR2

NCSTN

NOTCH1

NOTCH2

NOTCH3

NOTCH4

NUMB

NUMBL

PSEN1

PSEN2

PSENEN

PTCRA

RBPJ

RBPJL

RFNG

SNW1

AGO1

AGO2

AGO3

AGO4

ATP2A1

ATP2A2

ATP2A3

B4GALT1

CCND1

E2F1

E2F3

ELF3

FURIN

H2AB1

H2AC14

H2AC20

H2AC4

H2AC6

H2AC7

H2AC8

H2AJ

H2AX

H2AZ1

H2AZ2

H2BC1

H2BC10

H2BC11

H2BC12

H2BC13

H2BC14

H2BC15

H2BC17

H2BC21

H2BC3

H2BC4

H2BC5

H2BC6

H2BC7

H2BC8

H2BC9

H2BS1

H2BU1

H3-3A

H3-3B

H3C1

H3C10

H3C11

H3C12

H3C13

H3C14

H3C15

H3C2

H3C3

H3C4

H3C6

H3C7

H3C8

H4-16

H4C1

H4C11

H4C12

H4C13

H4C14

H4C15

H4C2

H4C3

H4C4

H4C5

H4C6

H4C8

H4C9

JUN

MAMLD1

MIR150

MIR181C

MIR200B

MIR200C

MIR206

MIR302A

MIR34B

MIR34C

MIR449A

MIR449B

MIR449C

MOV10

POFUT1

POGLUT1

PRKCI

RAB6A

RUNX1

SEL1L

SIRT6

ST3GAL3

ST3GAL4

ST3GAL6

TFDP1

TFDP2

TMED2

TNRC6A

TNRC6B

TNRC6C

TP53

ACTA2

ADAM10

AKT1

APH1B

ARRB1

ARRB2

CCNC

CDK8

CNTN1

CREB1

CUL1

DLGAP5

DLK1

DNER

EGF

EGFR

FABP7

FBXW7

FCER2

FLT4

GZMB

HDAC10

HDAC11

HDAC3

HDAC4

HDAC5

HDAC6

HDAC7

HDAC8

HDAC9

HEY1

HEY2

HEYL

HIF1A

IKZF1

ITCH

MDK

MIB1

MIB2

MYC

NBEA

NCOR1

NEURL1

NEURL1B

PBX1

PLXND1

PSMA1

PSMA2

PSMA3

PSMA4

PSMA5

PSMA6

PSMA7

PSMB1

PSMB10

PSMB2

PSMB3

PSMB4

PSMB5

PSMB6

PSMB7

PSMB8

PSMB9

PSMC1

PSMC2

PSMC3

PSMC4

PSMC5

PSMC6

PSMD1

PSMD10

PSMD11

PSMD12

PSMD13

PSMD14

PSMD2

PSMD3

PSMD4

PSMD5

PSMD6

PSMD7

PSMD8

PSMD9

PSME1

PSME2

PSME3

PSMF1

RBX1

RPS27A

SEM1

SKP1

SMAD3

STAT1

TACC3

TBL1X

TBL1XR1

TLE1

TLE2

TLE3

TLE4

UBA52

UBB

UBC

WWC1

WWP2

YBX1

YWHAZ

ADAM12

CBL

CDKN1A

CNTN6

DNM1

ENO1

EPS15

GATA3

IL4

KDM1A

LNX1

MARK2

MFAP2

MFAP5

MYCBP

RAB11A

RBBP8

SKP2

SPEN

SSPOP

YY1

FBXW11

FZD1

FZD5

FZD7

PPARD

PRKCA

SAP30

TCF7L2

WNT2

WNT5A

FHL1

GSK3B

HES6

JAK2

LCK

MAGEA1

MAPT

MIR1281

NFKB1

PIK3R1

PIK3R2

RING1

SRC

STAT3

AAK1

AGXT

ANGPT4

ANXA4

APP

ARRDC1

ASCL1

ATOH1

BCL6

BEND6

BLOC1S2

BMP2

BMP2K

BMP7

CBFA2T2

CCN3

CD46

CDH6

CDK3

CDK6

CDKN1B

CEBPA

CFAP58

CFD

CHAC1

DLK2

DLX1

DLX2

EGFL7

ENHO

EPN1

EPN2

ETV2

FGF10

FOXA1

FOXC1

FOXC2

GALNT11

GAS2

GATA2

GATA5

GMDS

GOT1

GRIP2

GSX2

HES3

HES7

HIF1AN

HNF1B

HOXD3

IFT172

IFT74

IL17A

IL2RA

IL6ST

ITGB1BP1

KCNA5

KIT

KRT19

LLGL1

LLGL2

MESP1

MESP2

METTL3

MIR1224

MIR126

MIR212

MMP14

NEPRO

NEUROD4

NFKBIA

NIBAN2

NKAP

NLE1

NOD2

NOS3

NOTCH2NLA

NOTCH2NLB

NOTCH2NLC

NR0B2

NR1H4

NRARP

ONECUT1

PDCD10

PERP

PGAM2

PLN

POSTN

PRAG1

PTP4A3

RBM15

RIPPLY2

RITA1

ROBO1

ROBO2

S1PR3

SIX1

SLC35C1

SLC35C2

SNAI1

SNAI2

SORBS2

SOX9

SREBF2

SUSD5

SYNJ2BP

TBX2

TCIM

TGFB2

TGFBR2

TIMP4

TM2D3

TMEM100

TP63

TRAF7

TSPAN14

TSPAN15

TSPAN5

TSPEAR

WDR12

WNT1

YAP1

YJEFN3

YTHDF2

ZBTB7A

ZMIZ1

ZNF423
